# Epithelial to Mesenchymal Transition and Cell Biology of Molecular Regulation in Endometrial Carcinogenesis

**DOI:** 10.3390/jcm8040439

**Published:** 2019-03-30

**Authors:** Hsiao-Chen Chiu, Chia-Jung Li, Giou-Teng Yiang, Andy Po-Yi Tsai, Meng-Yu Wu

**Affiliations:** 1 Department of Obstetrics and Gynecology, Taipei Tzu Chi Hospital, Buddhist Tzu Chi Medical Foundation, Taipei 231, Taiwan; 97311141@gms.tcu.edu.tw; 2 Department of Obstetrics and Gynecology, School of Medicine, Tzu Chi University, Hualien 970, Taiwan; 3 Department of Obstetrics and Gynecology, Kaohsiung Veterans General Hospital, Kaohsiung 813, Taiwan; nigel6761@gmail.com; 4 Department of Emergency Medicine, Taipei Tzu Chi Hospital, Buddhist Tzu Chi Medical Foundation, New Taipei 231, Taiwan; gtyiang@gmail.com; 5 Department of Emergency Medicine, School of Medicine, Tzu Chi University, Hualien 970, Taiwan; 6 Department of Medical Research, Buddhist Tzu Chi General Hospital, Hualien 970, Taiwan; tandy@iu.edu

**Keywords:** endometrial cancer, epithelial-mesenchymal transition, AKT/PI3K, Ras/Raf/MEK/ERK, WNT/β-catenin

## Abstract

Endometrial carcinogenesis is involved in several signaling pathways and it comprises multiple steps. The four major signaling pathways—PI3K/AKT, Ras/Raf/MEK/ERK, WNT/β-catenin, and vascular endothelial growth factor (VEGF)—are involved in tumor cell metabolism, growth, proliferation, survival, and angiogenesis. The genetic mutation and germline mitochondrial DNA mutations also impair cell proliferation, anti-apoptosis signaling, and epithelial–mesenchymal transition by several transcription factors, leading to endometrial carcinogenesis and distant metastasis. The PI3K/AKT pathway activates the ransforming growth factor beta (TGF-β)-mediated endothelial-to-mesenchymal transition (EMT) and it interacts with downstream signals to upregulate EMT-associated factors. Estrogen and progesterone signaling in EMT also play key roles in the prognosis of endometrial carcinogenesis. In this review article, we summarize the current clinical and basic research efforts regarding the detailed molecular regulation in endometrial carcinogenesis, especially in EMT, to provide novel targets for further anti-carcinogenesis treatment.

## 1. Introduction

Endometrial cancer is the most common neoplasm of the female genital tract. Its incidence and mortality rates are increasing. Endometrial carcinogenesis is a complex and multi-step process that features a slow progression from hyperplasia to endometrial cancer [1,2]. Several risk factors have been implicated and investigated, especially obesity and diabetes mellitus. As summarized by Sanderson et al. [3], the factors that contribute to endometrial carcinogenesis during estrogen stimulation include polycystic ovary syndrome (PCOS), obesity, perimenopause, functional tumor, and iatrogenic events. These conditions induce continuous estrogen stimulation that is unopposed by progesterone. Features of PCOS also include obesity and hyperinsulinemia, which also are important risk factors in endometrial carcinogenesis. Estrogen overstimulation was also associated with the phosphoinositide 3-kinase/protein kinase B (PI3K/AKT) pathway and the downstream mammalian target of rapamycin (mTOR) signaling to promote epithelial-mesenchymal transition (EMT), which occurs due to the inhibition of E-cadherin [4].

Genetic mutations also introduce several functional abnormalities and increased stress, which promote carcinogenesis. Kandoth et al. [5] collected and analyzed 373 high-grade endometrioid tumors. Mutations in *PTEN, CTNNB1, PIK3CA, ARID1A, KRAS*, and *ARID5B* were detected at high frequencies by array and sequencing analyses. *PTEN* and *KRAS* mutations in endometrial carcinoma may trigger the PI3K/AKT and mitogen-activated protein kinase/extracellular signal-activated kinase (MAPK/ERK) pathway. *PIK3CA* and *PIK3R1* mutations frequently occur with the *PTEN* mutation. *KRAS* and *CTNNB1* mutations are also involved in WNT signaling in endometrial carcinogenesis. Chang et al. [6] investigated genomic alterations in 14 tumor tissues from Taiwanese endometrial cancer patients. The authors reported nine potential driver genes (*MAPT, IL24, MCM6, TSC1, BIRC2, CIITA, DST, CASP8,* and *NOTCH2*) and 21 potential passenger genes *(ARMCX4, IGSF10, VPS13C, DCT, DNAH14, TLN1, ZNF605, ZSCAN29, MOCOS, CMYA5, PCDH17, UGT1A8, CYFIP2, MACF1, NUDT5, JAKMIP1, PCDHGB4, FAM178A, SNX6, IMP4*, and *PCMTD1)* and impaired cell functions that included cell proliferation, cell cycling, and death, via the mTOR, Wnt, MAPK, and vascular endothelial growth factor (VEGF) pathways. Gibson et al. [7] analyzed 98 tumor tissues using whole-exome sequencing. The mutation of *NRIP1*, which is an obligate cofactor of the estrogen receptor, accounted for 12.5% of the mutations.

However, detailed pathophysiology of endometrial cancer has remained unclear in clinical or basic studies. Many studies have focused on the molecular and cell biology on endometrial cancer, including the immune escape, local inflammation, mitochondria dysfunction, tumor cell proliferation, and cell death. These studies have informed novel approaches for the therapeutic strategies of endometrial cancer [8]. In this review, we present recent evidence and summarize the current concept of cell biology and molecular regulation of endometrial cancer. Our aim is to provide a strong foundation for the development of further therapeutic interventions.

## 2. Clinical Feature of Endometrial Carcinoma

Endometrial cancer is the seventh most common cancer globally, with increasing rates of incidence rate and mortality. An incidence of 24.7 per 100,000 women has been reported in Flanders and similar rates have been reported in other western European countries [9,10]. The typical presentation in endometrial cancer is abnormal uterine bleeding, especially in postmenopausal women. Abnormal uterine bleeding has been reported in about 60% of endometrial cancer patients [11,12] In some cases, due to atypical presentation the endocervical cavity anomaly may delay the diagnosis of endometrial cancer [13]. Other incidental findings of endometrial cancer may be obtained from cervical cytology or image findings. In cervical cytology analysis, adenocarcinoma was reported to arise from the cervix or endometrium, and further survey is necessary [14,15]. The presence of atypical glandular cells and endometrial cells in high risk patients is a hint to physicians to assess the endometrial neoplasm. In an update, Sanderson et al. [3] described the necessity for initial management of endometrial hyperplasia in abnormal uterine bleeding cases. In an atypical hyperplasia (AH) group, total hysterectomy with or without bilateral salpingo-oophorectomy (BSO) was suggested. If fertility is an issue or for patients in whom surgery is contraindicated, knowledge regarding the risk factors that are present is important in the control of estrogen stimulation. The Levonorgestrel-releasing intrauterine system (LNG-IUS) is the first-line therapy for these patients. In the endometrial hyperplasia without atypia group (EH), addressing the risk factors has also been suggested as a first step. Endometrial biopsy every six months in EH and three months in AH is prudent. The results are divided into three groups: regression, persistence, and prognosis. In regression cases, the continuation of LNG-IUS for five years was suggested, and oral progestogen may stop after six months. In the persistence group, 12 months of medical treatment was suggested in EH patients. Total hysterectomy with BSO was advised in AH patients with persistence and progression in pathological reports [16].

Transvaginal ultrasound (TVU) is an effective and noninvasive method in assessing the thickness and the characteristics of endometrium. The sensitivity and specificity of TVU with a cut-off of 5 mm were reported as 80.5% and 85.7% [17,18]. Computed tomography (CT) is an alternative tool to assess huge tumors in the pelvis [19]. However, the resolution of soft tissue by CT is lower when compared to magnetic resonance imaging (MRI). MRI can provide detailed information regarding tumor invasion and lymphadenopathy in endometrial cancer patients [20]. Preoperative staging via preoperative assessments, including TVU, CT for lung and liver, and MRI for retroperitoneal lymph nodes, is necessary for physicians to detect early stage or advanced disease [21,22]. In advanced disease, laparotomy, which prevents port-site metastasis, or palliative treatment, may be suitable for these patients [23]. However, endometrial cancer is a surgically staged disease, which comprises myometrial and intra-abdominal invasion. The International Federation of Gynecology and Obstetrics (FIGO) staging system is based on the myometrial and intra-abdominal invasion, such as the involvement of uterine serosa, adnexa, ascites, and intra-abdominal lymph nodes, to predict the mortality rate (Table 1). Previous studies reported that the FIGO staging system significantly reflected the five-year survival rate, with rates of 85% for stage I, 93% for stage IA, 90% for stage IB, 75% for stage II, 45% for stage III, and 25% for stage IV [24,25].

According to the clinicopathologic features, endometrial cancer is divided into two major types. Type 1 endometrial carcinomas include low-grade endometrioid endometrial carcinomas (FIGO grades 1 and 2), which accounted for the majority of cases (80% in one study [26]). Estrogen, from endometrial hyperplasia to the early stage induced these neoplasms. The prognosis is better than the prognosis for type 2 neoplasms, with FIGO grade 3 endometroid and nonendometrioid histologies that include serous, clear cell, mixed cell, and undifferentiated types. These type 2 neoplasms are not responsive to estrogen and they are associated with a poor prognosis. Endometrioid endometrial carcinoma can be induced by exposure to endogenous or exogenous estrogen, leading to abnormal endometrial proliferation, which causes endometrial adenocarcinoma. Several risk factors, including obesity and type 2 diabetes mellitus, reportedly promote the carcinogenesis of the endometrium. Genetic mutations, such as *PTEN, KRAS, ARID1A, PIK3CA,* and *CTNNB1* and microsatellite instability have been investigated [27]. These mutations and stress induced downstream pathways promote carcinogenesis [28]. Type 2 endometrial cancers, such as serous, clear cell carcinomas, and carcinosarcomas, are not associated with estrogen stimuli induced by different mechanisms. Mutations of p53 are commonly involved in the disease [29,30,31]. The detailed pathogenesis of endometrial carcinoma is still not well understood. Some of the mechanisms that have been implicated in the pathogenesis of endometrial carcinoma are considered in the following sections.

## 3. Signaling Pathways in Endometrial Carcinogenesis

**Figure 1:**
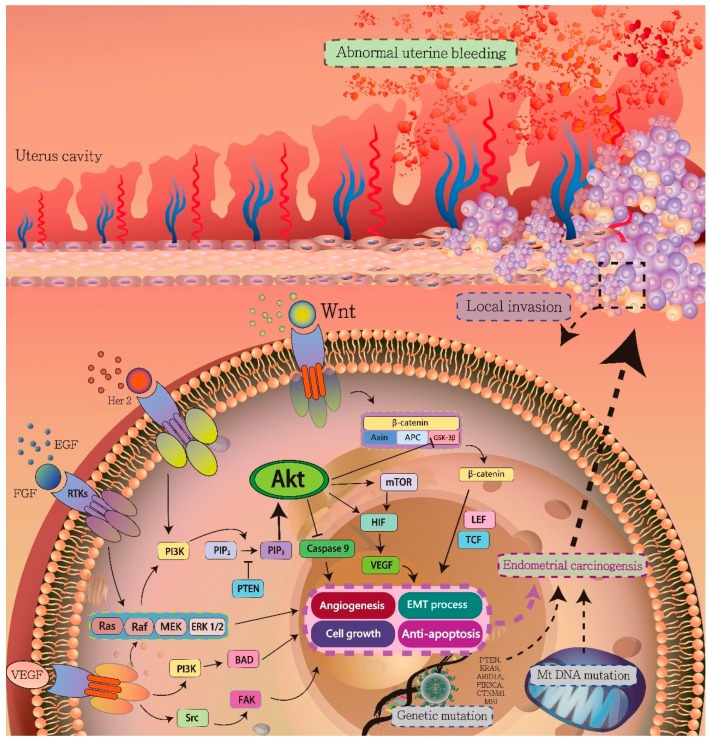
Schematic drawing presents the detail signaling pathways of endometrial carcinogenesis. The genes mutations and imbalance of estrogen and progesterone may triggered the several pathways, including Ras/Raf/MEK/ERK signaling pathway, Wnt/β-catenin signaling pathway, AKT/PI3K Pathway, vascular endothelial growth factor (VEGF) pathway, and mtDNA mutation, involved in carcinogenesis to induce cell proliferation, angiogenesis, epithelial-mesenchymal transition, and anti-apoptosis effect, promoting the cancer cell to local invasion and distant metastasis.

Endometrial carcinogenesis involves several signaling pathways that promote cell proliferation and facilitate the escape from the immune system and apoptosis signaling [32,33,34]. Several major pathways that were identified in endometrial cancer,, such as hypoxia-inducible factor 1 alpha (HIF-1α)/VEGF, PI3K/AKT/mTOR, Ras/Raf/MEK/ERK, Wnt/β-catenin, and Insulin/Insulin growth factor-1 (IGF-I) signaling pathways (Figure 1). Detailed knowledge regarding these signaling pathways is necessary to understand the pathophysiology of carcinogenesis for the development of novel targeted endometrial cancer therapies.

### 3.1. HIF-1α/VEGF Axis

Hypoxia is an important microenvironment in tumor growth and malignant progression. Hypoxia triggers the transcription of key genes for cell adaptation, including those that are important in angiogenesis and neovascularization, to increase oxygen availability, local invasion, and distant metastasis to escape from hypoxia and for metabolic reprogramming to adapt to hypoxia [35,36]. Hypoxia was initially commonly described in solid tumors, but further studies showed that hypoxia-inducible factor (HIF) is also involved in endometrial carcinogenesis [37,38,39]. The tumor has been associated with hypoxia, which stabilizes HIF-1α, which is a transcriptional activator of various genes, to upregulate VEGF levels and promote vascular growth and tumor progression. In a hypoxic environment, the stable form of HIF-1α acquires transactivation activity via the lowered activity of prolyl and asparagine dioxygenases. HIF-1α translocates to form a heterodimer with HIF-1β. HIF-1 induces the transcription of various genes by binding with HIF-responsive elements (Figure 1) [40]. VEGF is one of the important genes that is involved in endometrial carcinogenesis [41]. The VEGF levels are high and microvessel proliferation (MVP) is extensive in poorly differentiated, advanced, and metastatic endometrial cancer specimens. This clinical feature also reveals an important role for increased angiogenesis in the progression of endometrial cancer [42]. Endometrial cancer is a hypoxic tumor that occurs in endometrial epithelial tissue in peri-menopausal and postmenopausal women. In an anoxic environment, endometrial cancer cells form a new vascular system, which is an important mechanism in the adaptation to hypoxia [37,43].

VEGF is a key cell growth factor that stimulates tumor blood vessel growth [44]. Endometrial cancer is crucial in the expression of VEGF during the formation of new tumor blood vessels. However, HIF-1 directly regulates the increased expression of VEGF [45,46]. The regulation mechanism and clinical application of VEGF in endometrial cancer have become important topics of study [40]. In addition, estrogen may activate the nuclear factor-kappa B pathway by activating the P13K/AKT pathway to produce VEGF factor, which in turn promotes the proliferation and migration of endometrial cancer cells [47,48]. P13K/mTOR pathway inhibitors can sensitize endometrial cancer to radiation therapy by inhibiting HIF-1a/VEGF signaling (Figure 1) [49].

Angiogenesis plays a vital role in the pathological development of tumors. Due to the sustained action of VEGF, a large number of new blood vessels can be produced in the progression of endometrial cancer. The new blood vessels not only provide the tumor tissue with the nutrients necessary for growth, but they also remove metabolic products [50,51]. As a major regulator of neovascularization in endometrial cancer, HIF-1 directly regulates the expression of VEGF at the gene level. HIF-1α and VEGF are also closely related to early lymphatic metastasis of endometrial cancer [52]. Clinical pathological analysis has confirmed that lymphangiogenesis and lymphatic metastasis are early events in the dissemination of most solid tumors [53,54]. VEGF induces tumor lymph angiogenesis and it is an important cause of tumor cell metastasis through the lymphatic system [55]. In endometrial cancer tissues, the lower the degree of differentiation and the later the stage, the stronger is the positive expression rate of HIF-1α and the positive correlation with VEGF expression, suggesting that HIF-1α promotes angiogenesis by the regulation of the target gene, VEGF [56,57].

### 3.2. PI3K/AKT Pathway

The PI3K/AKT signaling pathway is a central regulator in endometrial carcinogenesis. The pathway connects intracellular and extracellular signaling [58]. The PI3K/AKT pathway reportedly mediates cell metabolism, growth, proliferation, survival, and angiogenesis [59]. In endometrial carcinogenesis progress, environmental stress and molecular alterations that result from growth factors, cytokines, insulin, and other factors induce PI3K signals by binding to the cell membrane receptors. The binding activates PI3K, which is then transformed to phosphatidylinositol 4,5-bisphosphate (PIP2), phosphatidylinositol 3,4,5-trisphosphate (PIP3), and phosphoinositide-dependent kinase 1 (PDK1). PDK1 phosphorylates the AKT protein [57,60], which inactivates the tuberous sclerosis complex (TSC) complex, which in turn leads to the downstream activation of mTORC1 [61]. The mTOR pathway was reported to activate endometrial carcinogenesis. In addition, signaling by receptor tyrosine kinases (RTKs) also induces the activation of the Ras/Raf/MEK/ERK signaling pathway (Figure 1) [62,63]. This pathway induces cell growth, proliferation, survival, and angiogenesis. The high frequency of the mutation of PIK3CA, a key gene that encodes the PI3K alpha subunit, has been described in endometrial cancer [64,65,66]. PTEN mutations are also frequently observed and they are present in up to 50% of endometrial cancers, followed by mutations of PIK3CA (30%) and K-Ras (20%) [64]. Mutations in PTEN may dysregulate PI3K/AKT/mTOR activation to inhibit apoptosis-related factors and activate anti-apoptotic factors, promoting endometrial carcinogenesis [67,68].

### 3.3. Ras/Raf/MEK/ERK Signaling Pathway

Multiple steps comprise the Ras/Raf/MEK/ERK signaling pathway. The pathway regulates cell proliferation and differentiation [69]. The signaling cascade is activated by several upstream signaling sources, which include genetic alterations, growth factors, cytokines, interleukin, and mitogen. These factors may interact with membranous receptors, such as RTKs, in endometrial cancer [70]. The activated Ras protein interacts with RAF to promote RAF phosphorylation and activate MEK. The activated MEK phosphorylates MAPK, which is also known as ERK [71]. MARK regulates cell proliferation and differentiation by mediating apoptosis signaling and cell cycle progression due to its interaction with the p53 pathway (Figure 1). In endometrial cancer, Ras/Raf/MEK/ERK signaling is often activated by the overexpression of receptors [72]. The mutation of the *K-RAS* gene, which encodes a small GTPase superfamily protein, has been frequently observed in 20% of endometrial cancers [64,73]. In addition, a similar mutation rate of *K-RAS* was noted in endometrial hyperplasia, when compared to endometrial carcinomas. The *K-RAS* mutations were as early events and were significantly associated with endometrial carcinogenesis [74].

### 3.4. Wnt/β-Catenin Signaling Pathway

The Wnt/β-catenin signaling pathway is divided into two major pathway types—the canonical (β-catenin dependent pathway) and the non-canonical pathway (Wnt/JNK or Wnt/calcium pathway)—which regulate several aspects of cell biology, including embryogenesis, cell differentiation, proliferation, and tumorigenesis [59]. Along with the Wnt ligand, Wnt interacts with the Frizzled family of proteins to inhibit the complex of APC/AXIN/CK1/GSK3β, which mediates the stabilization of β-catenin, and then it activates the translocation of β-catenin to the nucleus [75,76,77]. In the nucleus, β-catenin interacts with the T-cell factor/lymphoid enhancer-binding factor (TCF/LEF) family of transcription factors. This interaction preludes gene transcription that promotes cell proliferation and survival. In the non-canonical Wnt pathway, Wnt mediates the release of calcium and calmodulin by binding to the Frizzled receptors [78]. The non-canonical and canonical pathways interact with each other. Crosstalk and overlapping Wnt signaling pathways are features of the Wnt network theory [79]. The Wnt/β-catenin signaling is important in endometrial hyperplasia and cancer (Figure 1). Mutations in the gene encoding β-catenin gene mutations produced an accumulation of β-catenin in 38% of endometrial carcinoma cases in one study, and immunohistochemistry subsequently demonstrated the nuclear accumulation of β-catenin in 12–31% of the endometrial cancer cases examined [80,81]. The overexpression of Wnt pathway components, such as Wnt7a, has been correlated with advanced tumor progression and poor prognosis in endometrial cancer [82,83,84]. Progesterone and estrogen are also involved in Wnt signaling and they regulate the endometrial cycle [85]. These two hormones establish a dynamic balance that regulates the menstrual cycle. During the first two weeks of the menstrual cycle, the release of large amounts of estradiol from the cells triggers an increase in the levels of the endometrial estrogen receptor (ERα). The ER signaling promotes PI3K/AKT, MAPK, and Wnt signaling, which induce endometrial proliferation via the transcription of the downstream target genes [86,87,88,89]. During the late stage of the menstrual cycle, the release of progesterone by the corpus luteum inhibits estradiol control of the proliferation of endometrial cells. Endometrial carcinogenesis features an imbalance of the amounts of progesterone and estrogen. The overexpressed estrogen binds to ER-α, ER-β, and G protein-coupled estrogen receptor (GPR) 30, which triggers the over-transactivation of numerous growth-promoting genes, including epidermal growth factor (EGF), IGF-1, VEGF, and fibroblast growth factor (FGF). The over-activity leads to tumorigenesis. The dysfunction of progesterone in endometrial cancer has been reported [90]. In Ishikawa cells, progesterone may activate the expression of Dickkopf1 (DKK1) and Forkhead box protein O1 (FOXO1) to inhibit Wnt signaling [85]. Small interfering RNA-mediated knockdown of DKK1 and FOXO1 and immunohistochemical analysis have shown similar results and confirmed that progesterone inhibits Wnt signaling in endometrial hyperplasia and endometrial carcinogenesis. In stage I endometrial cancer, the expression of DKK1 and FOXO1 may be reduced due to poorly differentiated ER-α and the progesterone receptor (PR).

### 3.5. Insulin/IGF-I Signaling Pathway

Insulin/IGF-I activates the P13K/AKT signaling pathway through the tyrosine kinase receptor. This promotes downstream cyclinD1 expression and cell proliferation [91]. The observations that streptozotocin can reduce the circulating insulin levels in obese mice, and that the pro-proliferative effect of estrogen is significantly reduced, favor the suggestion that insulin may increase the estrogen sensitivity of the endometrial cells [92]. The changes in endometrial estrogen sensitivity may be associated with estrogen receptor levels. IGF-I can upregulate the expression of the G protein-coupled estrogen receptor (GPER) in endometrial cancer cells and promote cell migration and proliferation [93]. In addition, the IGF-I receptor can be overexpressed in endometrial proliferative lesions and tumor suppressor genes, and PTEN deficiency has been associated with a frequent incidence of endometrial proliferative lesions [94]. The collective findings indicate that the insulin/IGF-I signal can cooperate with other signals to play a role in promoting cancer.

## 4. Epithelial-Mesenchymal Transition (EMT) Signaling Pathways in Endometrial Carcinoma

### 4.1. Classification of EMT

EMT is an important process, in which epithelial cells lose their cell–cell contacts and adopt a mesenchymal-like property that features cytoskeleton remodeling and migratory activity [63]. EMT is important in embryonic development and tissue repair [95]. There are three types of EMT. Type 1 is responsible for embryogenesis and organ development. Cells can differentiate to form different types of secondary epithelial tissues [96]. Type 2 is induced by inflammation and fibrosis for tissue regeneration and organ fibrosis. In carcinogenesis, type 3 EMT reportedly explains the transformation of secondary epithelial cells into cancer cells and the resulting local invasion and distant metastasis [97,98]. Epithelial cell transformation to invasive cancer cells is a complex and multi-stage process. Initially, the polarity of epithelial cells is lost, leading to cell detachment from the basement membrane due to the changes of extracellular matrix (ECM) interactions [99]. EMT is involved in the tumor growth phase and the progression to metastatic cancer. The EMT signaling pathways may be activated by several cytokines or growth factors from the local microenvironment, followed by the interaction with transforming growth factor beta (TGF-β), bone morphogenetic protein, Wnt/β-catenin pathway, Notch, Hedgehog, and RTKs [100,101]. The involvement of EMT in the carcinogenesis process in endometrial cancer was recently demonstrated, which involved E-cadherin loss or the induction of its repressors. Other gynecologic cancers also feature the involvement of the EMT pathway [102].

### 4.2. Effect of Tumor Microenvironment on TGF-β-Mediated EMT

Normal cells and cancer cells respond differently to transforming growth factor beta (TGF-β) in different ECMs [103]. TGF-β regulates the activation of cancer-associated fibroblasts (CAF) around cells and myofibroblasts. CAFs secrete numerous pro-tumorigenic cytokines that include interleukin-6 (IL-6), which can be isolated from endometrial cancer tissues and promote cell proliferation [104]. CAFs are also involved in endometrial carcinogenesis due to their secretion of IL-6, IL-8, MCP-1, CCL5, and RANTES to promote cancer progression [105]. Subramaniam et al. [106] described the primary culture of CAFs that was obtained from human endometrial cancer tissues by antibody-conjugated magnetic bead isolation. The CAFs displayed specific effects that triggered endometrial cancer cell proliferation, as compared to fibroblasts that were cultured from benign endometrial hyperplasia tissues. The CAFs induced the proliferation of endometrial cancer cells by the SDF-1α/CXCR4 axis, which activated the PI3K/AKT and MAPK/ERK signaling pathways via paracrine hormone (Figure 1). The effect also promoted matrix metalloproteinase 2 (MMP-2) and MMP-9 secretion via autocrine the hormone [107]. The mTOR signaling was reported in CAF-mediated cell proliferation and it was confirmed by the use of the mTOR inhibitor rapamycin [106,108]. These results were not found in normal endometrial fibroblasts. When these cells were activated, they can synthesize and secrete a variety of growth factors, chemokines, and ECM proteins, which can promote the carcinogenesis of the adjacent epithelial cells [99,106,109]. In vitro experiments have demonstrated that these cells can be induced to exhibit cancer-like structures by selectively blocking the expression of type II TGF-β receptor (TβR-II) in prostate and gastric mesenchymal fibroblasts [110]. By inhibiting the activation of CAF, TGF-β can exert a tumor suppressor effect. The loss of the TGF-β signal in CAFs is an environmental factor that promotes the carcinogenesis and metastasis of CAFs.

### 4.3. Transcriptional Regulators in TGF-β and EMT

The EMT pathway is triggered by several transcription factors in different signaling pathways. TGF-β is the major factor that leads to EMT via SMAD-mediated and non-SMAD signaling. Both endometrial cancer and stromal cells produce high levels of TGF-β and they may recruit TGF-β secreting cells, including macrophages and neutrophils [111]. The release of TGF-β from epithelial cancer cells can also regulate the microenvironment of the tumor mass via an autocrine or paracrine [112]. TGF-β binding to membrane receptors activates SMAD2/3 to bind SMAD4 and form the SMAD complex [100]. By promoting EMT, this complex is involved in the regulation of transcription following its translocation into the nucleus (Figure 1) [113]. TGF-β has several roles in the EMT process, which include the activation of epithelial proteins expression via EMT transcription factors, reducing the expression of the epithelial splicing regulatory proteins, and increasing the activity of non-SMAD signaling pathways, including the PI3K/AKT pathway for translational regulation, partitioning defective 6 (PAR6) complex for cell junction dissolution, and the activity of RHOA, RAC, and CDC42 for cytoskeletal changes. In endometrial carcinogenesis, the imbalance of TGF-β signaling at the early stages is a key signal, which causes abnormal proliferation of the endometrium [114]. In a microarray gene expression analysis study, TGF-β1 induced EMT signaling to promote an invasive phenotype in HEC-1A and RL95-2 cells [113]. After administration of the TGF-β1 inhibitor, SB-431542, the endometrial carcinoma invasion was controlled and precluded [113,115]. Additionally, the overexpression of microsatellite instability dominant-negative RII (DNRII) blocked TGF-β signaling in human endometrial carcinoma HEC-1-A cells, which significantly inhibited cell proliferation and growth and stimulated apoptosis. The DNRII cells also showed more epithelial features and they were reduced in their capacity to migrate, invade, and metastasize, when compared to the control group [115].

TGF-β signaling has two major functions—the activation of SMAD signaling and non-SMAD pathways. Several non-SMAD signaling pathways have been implicated in the response to the full EMT process, including the PI3K/AKT, Ras/Raf/MEK/ERK, and Wnt/β-catenin signaling pathways (Figure 1) [116,117]. TGF-β interacts with the TGF-β receptor type I membrane receptor to phosphorylate the adaptor protein SRC homology 2 domain-containing-transforming A (SHCA). The phosphorylation leads to the activation of the Ras/Raf/MEK/ERK signaling pathway via the growth factor receptor-bound protein 2 (GRB2) and son of sevenless (SOS). TGF-β signaling also promotes the p38 and JNK activation by tumor necrosis factor receptor-associated factor 6 (TRAF6) to process EMT signaling. TGF-β signaling and several growth factors, such as VEGF, EGF, FGF, and hepatocyte growth factor, via RTKs induce the Ras/Raf/MEK/ERK signaling pathway. Wnt signaling is activated, which results in the release of β-catenin into the nucleus by the inhibition of glycogen synthase kinase-3β (GSK3β). β-catenin interacts with LEF and TCF, leading to the EMT process. Other signaling pathways, including the Notch and Hedgehog pathways, also promotes EMT in endometrial carcinogenesis by the induction of SNAIL1 expression, which leads to decreased local levels of E-cadherin. EMT promoted by SNAIL, and zinc-finger E-box-binding (ZEB) and basic helix–loop–helix (bHLH) transcription factors cause the downregulation of epithelial marker genes and the establishment of a mesenchymal phenotype. In endometrial cancer, a decreased level of E-cadherin was reported in 19.5% of G1, 40.8% of G2, and 72.7% of G3 of endometrial cancer [118] with an elevation of Snail and Slug nuclear expression. In addition, clinical outcomes, such as histological type, FIGO stage, local invasion, and cytology, have been strongly associated with the expression of E-cadherin and Snail, which significantly reflect the EMT status, and are prognostic factors in endometrial cancer [118].

### 4.4. Estrogen Signaling in EMT

Estrogen signaling is an important prognostic marker that reflects the resistance to hormonal therapies in endometrial cancer. Non-genomic and genomic responses may trigger the estrogen signaling pathways [119]. In the genomic pathway, estrogen directly binds estrogen receptor alpha (ERα) to form the ER-E complex. After dimerization, the ER-E complex may directly mediate gene expression or interact with transcription factors to regulate gene expression [120]. In the non-genomic pathway, the ER-E complex forms in the membrane, where it binds the proto-oncogene tyrosine-protein kinase Src. This triggers calcium release and induces the protein kinase A (PKA) pathway. PKA signaling regulates the expression of estrogen-responsive genes through the activation of transcription factors. These genetic expressions promote cell proliferation. ERα also closely regulates progesterone signaling. Mohammed et al. [121] described that the progesterone receptor is an ERα-induced target gene that also modulates ERα behavior. The PR-A and PR-B ligands can inhibit estradiol-stimulated ER activity to control the progression of endometrial cancer [122]. Estrogen also induces another non-transcriptional regulation pathway to over-activate the PI3K/AKT pathway. The in vitro results confirmed that the PI3K/AKT signal pathway is activated in Ishikawa cells via an ER-dependent pathway and in HEC-1A cells via an ER-independent pathway [123]. Estrogen stimulation in type I and II endometrial cancer has also been associated with the PI3K/AKT pathway via the binding to p85/p110 [124]. Downstream of the PI3K signaling pathway activates mTOR signaling, leading to the transcription of Snail 1/2 and Twist and the promotion of EMT via the inhibition of E-cadherin [4]. Poor estrogen signaling expression has been correlated with an advanced phenotype, especially in type II endometrial carcinoma [4]. In addition, the reduced expression of estrogen signaling has been associated with the increased expression of multiple EMT markers, including Snail1/2, Twist, and ZEB 1/2. Blocking Akt signaling by PI3K inhibitors may provide a new target in endometrial cancer.

GPER is a protein that is encoded by the GPER gene. The protein binds to estradiol with high affinity as the third estrogen receptor [125]. The binding of estrogen to GPER activates adenylyl cyclase to induce the activation of MMP and trigger the heparan bound EGF. These factors form the membrane-localized GPER-1, which promotes MAPK and PI3K signaling. Immunohistochemical studies have significantly associated GPER-1 expression with progression of female reproductive cancer. In the HEC50 endometrial cancer cell line, estrogen signaling is activated through GPER to induce the downstream PI3K/AKT pathway [126]. This pathway promotes the EMT process and induces high-grade invasive endometrial cancer.

### 4.5. Progesterone Signaling in EMT

The expression of PRs has been reported as a key factor that is associated with prognosis and drug-resistance of endometrial cancer [127,128] The well-differentiated endometrial cancer usually presented with PR, which may maintain the effect of hormone therapy, such as medroxyprogesterone acetate. When the expression of PR is lost, the target for hormone therapy is also lost, which is a negative prognostic factor. This type endometrial cancer usually progresses to a more advanced and invasive phenotype. The effect of hormone therapy may only be successful in 15–20% of cases of invasive phenotype endometrial cancer [129,130]. In endometrial cancer, the loss of the expression of progesterone signaling may trigger the EMT and diminish T-cell infiltration [131]. Several signaling pathways have been identified in progesterone modulated cell lines, including EGF, IL-6, PDGF, TGF-β, VEGF, and Wnt/β-catenin signaling, which mediate gene expression that is associated with the progressive and non-progressive phenotypes. A strong link between progesterone and the TGF-β signaling pathway has been reported in many studies. The increased expression of TGF-β triggers endometrial cancer that features a poor survival rate. Progesterone has a chemoprotective effect in endometrial cancer by impairing TGF-β signaling. In vitro, progesterone significantly decreased TGF-β signaling 72 h after treatment in Ishikawa cells [117]. In the same study, progesterone also effectively inhibited endometrial cancer cell viability and invasion during the increased expression of E-cadherin. Thus, the progesterone signaling in endometrial cancer may be critical in stimulating immunosurveillance and inhibition of EMT [131].

## 5. Conclusions

In the present article, we have summarized the clinical and basic research that has been focused on the detailed molecular regulation of endometrial carcinogenesis. The PI3K/AKT pathway mediates cell metabolism, growth, proliferation, survival, and angiogenesis. It also activates Ras/Raf/MEK/ERK signaling, which in turn regulates cell proliferation and differentiation. The Wnt/β-catenin signaling pathway is also involved in endometrial carcinogenesis by regulating cell proliferation. These major signaling pathways collectively promote EMT by triggering several transcription factors. The epithelial cells lose cell–cell contacts and adopt a mesenchymal-like property, with cytoskeleton remodeling and migratory activity via the EMT process, which promotes endometrial carcinogenesis and distant metastasis. The mutations of mtDNA, especially germline mutations, are associated with tumorigenesis. The detailed mechanism of mtDNA mutation-involved in endometrial cancer is still unclear. These concepts are worthy of investigation to provide novel targets in the development of efficacious therapeutic interventions.

## Figures and Tables

**Table 1:** TNM and International Federation of Gynecology and Obstetrics (FIGO) staging scoring system of endometrial cancer.

When T	When N	When M	FIGO Stage
T1	N0	M0	I
T1a	N0	M0	IA
T1b	N0	M0	IB
T2	N0	M0	II
T3	N0	M0	III
T3a	N0	M0	IIIA
T3b	N0	M0	IIIB
T1-3	N1/N1mi/N1a	M0	IIIC1
T1-3	N2/N2mi/N2a	M0	IIIC2
T4	Any N	M0	IVA
Any T	Any N	M1	IVB

T: Extent of the primary tumor, N: Involved regional lymph nodes, M: Distant metastasis, N1mi/N2mi: nodal micrometastases.
